# Crosstalk between PML and p53 in response to TGF-β1: A new mechanism of cardiac fibroblast activation

**DOI:** 10.7150/ijbs.76214

**Published:** 2023-01-22

**Authors:** Di Huang, Dan Zhao, Ming Li, Si-Yu Chang, Ya-Dong Xue, Ning Xu, Si-Jia Li, Nan-Nan Tang, Li-Ling Gong, Yi-Ning Liu, Hang Yu, Qing-Sui Li, Peng-Yu Li, Jia-Li Liu, Hai-Xin Chen, Ming-Bin Liu, Wan-Yu Zhang, Xing-Miao Zhao, Xian-Zhi Lang, Zhen-Dong Li, Yu Liu, Zhi-Yong Ma, Jia-Min Li, Ning Wang, Hai Tian, Ben-Zhi Cai

**Affiliations:** 1 Department of Pharmacology at College of Pharmacy, Department of Pharmacy at the Second Affiliated Hospital, Harbin Medical University, Harbin, 150081, China.; 2 Research Unit of Noninfectious Chronic Diseases in Frigid Zone (2019RU070), Chinese Academy of Medical Sciences, Harbin, 150081, China.; 3 Northern Translational Medicine Research and Cooperation Center, Heilongjiang Academy of Medical Sciences, Harbin Medical University, Harbin, 150081, China.; 4 Department of Cardiovascular Surgery, The Second Affiliated Hospital of Harbin Medical University, Harbin, 150081, China.; 5 Future Medical laboratory, The Second Affiliated Hospital of Harbin Medical University, Harbin, 150081, China.

**Keywords:** PML SUMOylation, TGF-β1, p53, cardiac fibrosis

## Abstract

Cardiac fibrosis is a common pathological cardiac remodeling in a variety of heart diseases, characterized by the activation of cardiac fibroblasts. Our previous study uncovered that promyelocytic leukemia protein (PML)-associated SUMO processes is a new regulator of cardiac hypertrophy and heart failure. The present study aimed to explore the role of PML in cardiac fibroblasts activation. Here we found that PML is significantly upregulated in cardiac fibrotic tissue and activated cardiac fibroblasts treated with transforming growth factor-β1 (TGF-β1). Gain- and loss-of-function experiments showed that PML impacted cardiac fibroblasts activation after TGF-β1 treatment. Further study demonstrated that p53 acts as the transcriptional regulator of PML, and participated in TGF-β1 induced the increase of PML expression and PML nuclear bodies (PML-NBs) formation. Knockdown or pharmacological inhibition of p53 produced inhibitory effects on the activation of cardiac fibroblasts. We further found that PML also may stabilize p53 through inhibiting its ubiquitin-mediated proteasomal degradation in cardiac fibroblasts. Collectively, this study suggests that PML crosstalk with p53 regulates cardiac fibroblasts activation, which provides a novel therapeutic strategy for cardiac fibrosis.

## Introduction

Ischemic heart diseases remain a leading cause of patient death worldwide 1. Cardiac fibrosis is a common pathological remodeling in response to myocardial infarction (MI), characterized by excessive accumulation of extracellular matrix proteins in the interstitium of the heart. Cardiac fibrotic scar during MI leads to both cardiac systolic and diastolic dysfunction and ultimately contributes to heart failure 2. Actually, proper fibroblast proliferation and extracellular matrix deposition is necessary for maintaining the structural integrity of the infarcted ventricle. However, excessive fibroblast activation will cause pathological remodeling in MI patients, thus contributing to the pathogenesis of heart failure 3. At present, there are still a lack of specific and effective therapeutic treatments for cardiac fibrosis. Therefore, a better understanding of the molecular mechanisms of cardiac fibrosis is crucial to identify new therapeutic targets for the prevention and treatment of heart failure following MI.

Promyelocytic leukemia protein (PML) is originally discovered as a major component of PML-RARα fusion oncoprotein in patients suffering from acute promyelocytic leukemia (APL) 4, 5. A multiprotein complex made up of PML called PML nuclear bodies (PML-NBs), a membrane-less subnuclear organelle, is involved in diverse biological processes 6. The small ubiquitin-like modifier (SUMOylation) is essential for the formation of PML-NBs, which stabilizes PML protein through the covalent modification of SUMO-1, SUMO-2 and SUMO-3 7-9. Previous studies have shown that the UBC9/PML/RNF4 axis-mediated SUMO pathway promotes the transition from cardiac hypertrophy to heart failure 10. However, the molecular and signaling mechanism of PML in cardiac fibrosis are still unclear.

P53 is a key regulator involved in a wide variety of biological responses including apoptosis, cell cycle, senescence, autophagy, metabolism, and DNA repair 11. A number of studies have demonstrated the ability of PML to recruit p53 as well as some of its specific regulators into PML-NBs, such as HIPK2, CHK2, CBP, Mdm2 and SIRT1 12. A recent study also showed that upregulated Mad1 displaces Mdm2 from PML, freeing it to ubiquitinate p53 and promoting tumor initiation and growth 13. However, the characteristics of PML combined with p53 in the process of myocardial fibrosis remain unknown. We carried out the present study to elucidate the role and underlying mechanisms of PML in response to TGF-β1-induced myocardial fibrosis and uncover the potential feedback loop of PML/p53 during this process.

## Materials and Methods

### Animal models

The procedures of all animal experiments were in compliance with the rules of the Institutional Animal Care and Use Committee of Harbin Medical University, as well as the NIH regulations (Guide for the Care and Use of Laboratory Animals). Male C57BL/6 mice (8 weeks old) were provided by the Experimental Animal Center of Harbin Medical University (Grade II). Mice were housed in a clean, pathogen-free facility at a controlled temperature (20-23°C) on a 12 h light-dark cycle. Mice were anesthetized by intraperitoneal injection of avertin (Sigma-Aldrich, St Louis, USA). We performed a permanent ligation of the left anterior descending artery (LAD) under sterile conditions to construct a model of myocardial infarction. Successful occlusion was monitored to confirm ischemic ST-segment elevation by echocardiography and the observation of an arising pale ischemic zone with weak activity. Sham group mice were subjected to the same procedures as the MI group, except LAD ligation, and served as surgical controls. The mice subsequently underwent echocardiography and were sacrificed 1 week after surgery.

### Masson's trichrome staining

The hearts were perfused and fixed in 4% paraformaldehyde for 48 h at 4°C and embedded in paraffin, then stained using a Masson Trichrome Stain kit (Sigma-Aldrich, St Louis, USA). The extent of myocardial fibrosis was determined by evaluating the proportion of positively stained fibrotic region to the total myocardium area using Image-Pro Plus.

### Primary culture of neonatal mouse cardiac fibroblasts (NMCFs)

Neonatal mouse cardiac fibroblasts were isolated from mice (1-2 days old). The myocardial tissue was digested in 0.25% trypsin for 2 h at 37°C. Cardiomyocytes and cardiac fibroblasts are separated based on their different adhesion times. P0 cardiac fibroblasts were cultured in DMEM medium with 10% FBS (Biological Industries, Haemek, Israel). When growing up to 70%-80% confluency, NMCFs were treated with TGF-β1 (5 ng/ml) for 6 h (PeproTech, USA, #AF-100-21C). NMCFs were also treated with 10 μM pifithrin-α, a p53 inhibitor (MedChem Express, USA, #HY-15484), or ginkgolic acid (10 μM) (MedChem Express, USA, #HY-N0077) at 1 h before TGF-β1 treatment. For the protein stability assay, 10 μg/ml cycloheximide (MedChem Express, USA, #HY-12320) was added at the indicated times.

### Transfection, plasmids and siRNA

Lipofectamine 2000 reagent (Invitrogen, Carlsbad, CA, USA) and X-tremeGENE siRNA transfection reagent (Roche, Basel, Switzerland) were used for transfection of plasmids or siRNA, respectively. The sequences of siRNA used in this study are listed in Table S1. After a 48-hour period of transfection, cells were harvested for further experiments.

### Cell viability assay

Cell viability was measured using the Cell Counting Kit‐8 (CCK‐8) assay kit (MedChem Express, USA, #HY-K0301). The cells were incubated with 10 µl CCK‐8 solution for 2 h at 37°C. The absorbance value of each well was measured by the microplate reader (BioTek, Richmond, USA).

### Co-immunoprecipitation (Co-IP) and Western blotting

Co-IP assays require lysing of the cellular samples using RIPA lysis buffer containing a protease inhibitor cocktail. Whole-cell lysates were then treated with desired antibodies. Afterward, magnetized protein A/G beads (MedChem Express, USA, #HY-K0202) were used to capture the target protein. SDS-PAGE was used to separate immunoprecipitates or whole-cell lysates, followed by transfer to nitrocellulose membranes (Pall Corporation, Mexico, USA). The membranes were incubated overnight at 4°C with the following primary antibodies: PML (1:1000; Merck Millipore, USA, #MAB3738), SUMO-1 (1:500; Abcam, USA, #ab32058), SUMO-2/3(1:500; Abcam, USA, #ab3742), p53 (1:1000; Proteintech, USA, #10442-1-AP), p-p53 (1:1000; Cell Signaling Technology, USA, #9284) and α-SMA (1:1000; Sigma, USA, #A2547), Ubiquitin (1:500; Santa Cruz Biotechnology, USA, #sc-8017), Alpha Tubulin (1:2000; Proteintech, USA, #66031l-1-lg) and GAPDH (1:10000; ABclonal, USA, #AC002). After one-hour incubation with the appropriate secondary antibodies, the membranes were scanned with the Odyssey infrared imaging system (LI-COR, Lincoln, NE, USA). The intensity of bands was quantified using Image-J software.

### Quantitative real-time PCR (qRT‑PCR)

Total RNA from cells and heart tissues were extracted using Trizol reagent (Invitrogen, Carlsbad, CA, USA). The cDNA was generated using a High-Capacity cDNA Reverse Transcription Kit (Toyobo, Japan). Quantifying target genes with qRT-PCR was performed on a 7500 Fast Real-Time PCR System (Applied Biosystems, Foster City, USA) using SYBR Green PCR Master Mix Kit (Roche, Basel, Switzerland). GAPDH was used as the reference gene. The primer sequences used in this study are listed in Table S2.

### Chromatin immunoprecipitation (ChIP) assay

ChIP assay was performed using a chromatin IP assay kit (Invitrogen, Carlsbad, CA, USA) according to the manufacturer's protocol. In brief, NMCFs were collected from 10 cm dishes and lysed in a lysis buffer. Immunoprecipitation was carried out overnight with purified anti-p53 antibody (1:200; Cell Signaling Technology, USA, #2524) or normal mouse IgG as a negative control. PCR was performed to determine the enrichment of protein-bound DNA. The primer sequences are listed in Table S3.

### Immunofluorescence staining

For immunofluorescent staining, tissue sections or cells were incubated overnight at 4°C with primary antibodies against PML (1:500; Merck Millipore, USA, #MAB3738), α-SMA (1:400; Affinity, USA, #AF1032) and p53 (1:200; RD Systems, USA, #AF1355). Nuclear counterstain was performed with DAPI (Sigma-Aldrich, St Louis, USA) for 15 min. All imaging was captured with a fluorescence confocal laser scanning microscope (Zeiss, Oberkochen, Germany).

### Proximity ligation assay

NMCFs grew on coverslips. After washing three times with PBS, cells were fixed with 4% paraformaldehyde for 15 min, and then permeabilized with 0.2% Triton X-100 for 5 min. Cells were then blocked with 3% BSA, and 0.1% Tween-20 produced by SSC for 1 h at room temperature. Cells were then incubated with primary antibody against p53 (1:500; Cell Signaling Technology, USA, #2524), and against PML (1:500; Novus, USA, #NB100-59787) overnight at 4°C. Then cells were incubated with pre-mixed PLA probe anti-goat minus and PLA probe anti-rabbit plus (Sigma-Aldrich, St Louis, USA) for 1 h at 37°C. The proximal ligation assay was conducted with the Duolink *In situ* Kit (Sigma-Aldrich, St Louis, USA) according to the manufacturer's instructions. Nuclei were stained with DAPI and images were captured with a fluorescence confocal laser scanning microscope (Zeiss, Oberkochen, Germany).

### Statistical analysis

Group data are presented as mean ± SEM. The statistical significance of differences between two groups was assessed by a two-tailed Student's t-test. Multiple-group comparisons were performed by one-way ANOVA followed by Tukey's analysis for comparisons of mean values. *P* < 0.05 was taken to indicate statistical significant. Our statistical analyses were performed using GraphPad Prism 9 (GraphPad Software Inc., San Diego, CA).

## Results

### Upregulation of PML in cardiac fibrosis following MI and TGF-β1-treated cardiac fibroblasts

**Figure 1:**
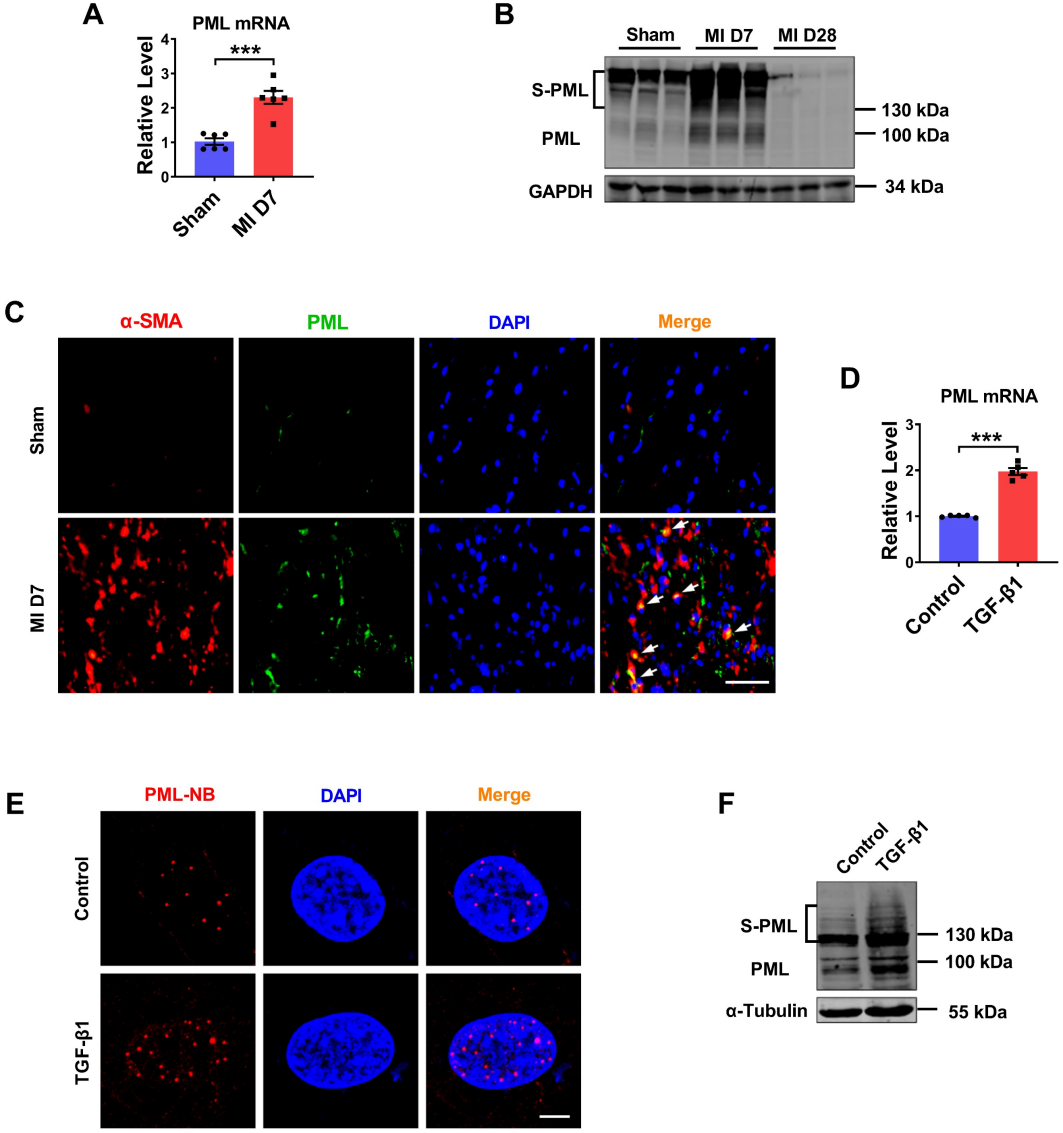
** Expression of PML in fibrotic heart tissue and cardiac fibroblasts treated with TGF-β1.** (**A**) qRT-PCR showing increased transcript level of PML in the infarct border zone of mouse left ventricular tissues at 7 days after MI operation (n = 6). (**B**) Western blot showing the expression of SUMOylated PML in the infarct border zone of mouse left ventricular tissues at 7 or 28 days after MI operation (n = 6). (**C**) IF staining of heart sections at 7 days after MI operation. Scale bar, 20 μm (n = 4). (**D**) Following treatment with TGF-β1, the mRNA level of PML in CFs was examined (n = 5). (**E**) PML-NBs in CFs were identified with immunofluorescence. Scale bar, 5 μm (n = 6). (**F**) SUMOylated PML protein in TGF-β1-treated CFs was visualized by western blot (n = 5). ***P < 0.001.

Cardiac fibroblasts proliferation and collagen deposition is a typical pathological process of MI injury and repair. To elucidate the role of PML in cardiac fibrosis, we analyzed the RNA-Seq data (GSE116250) in 64 samples from human left ventricle tissue: 14 nonfailing donors (NF), 37 dilated cardiomyopathies (DCM), and 13 ischemic cardiomyopathies (ICM) 14. The results showed that PML expression was significantly upregulated in patients with ICM compared with patients with nonfailing myocardium. In addition, the expression level of PML was more strikingly elevated in ICM than in DCM (Fig. S1A). Then we studied the changes of PML in mouse fibrotic tissues after MI (Fig. S1B-E). As shown in Fig. 1A, the mRNA level of PML was increased more than 2-fold in the MI mice. Meanwhile, we detected the protein expression of PML in the heart of mice at 7 and 28 days after MI. Western blotting showed that both unmodified PML (95 kDa) and the high-molecular-weight SUMO-modified PML (S-PML) (>130 kDa) were increased initially at 7 days and subsequently returned at 28 days post-MI (Fig. 1B). The data suggest that the upregulation of PML were associated with cardiac fibrotic response in infarcted hearts. Immunofluorescence staining demonstrated an increased expression of PML at the peri-infarct area in hearts post-MI compared with the sham group (Fig. 1C).

We also tested the changes of PML in cardiac fibroblasts (CFs) treated with TGF-β1. Consistent with *in vivo* results, TGF-β1 treated CFs present substantially increase of Col-1α1, Col-3α1 expression (Fig. S1F-G), as well as the increase of PML expression (Fig. 1D). Immunofluorescence performed in CFs further revealed that after treatment of TGF-β1, PML-NBs accumulated significantly in the nucleus (Fig. 1E). Immunoblotting analysis showed that SUMOylated PML accumulation was increased by TGF-β1 (Fig. 1F). These results revealed the upregulation of PML in cardiac fibrosis in MI and TGF-β1-treated cardiac fibroblasts.

### Pro-fibrotic action of PML on cardiac fibroblasts

**Figure 2:**
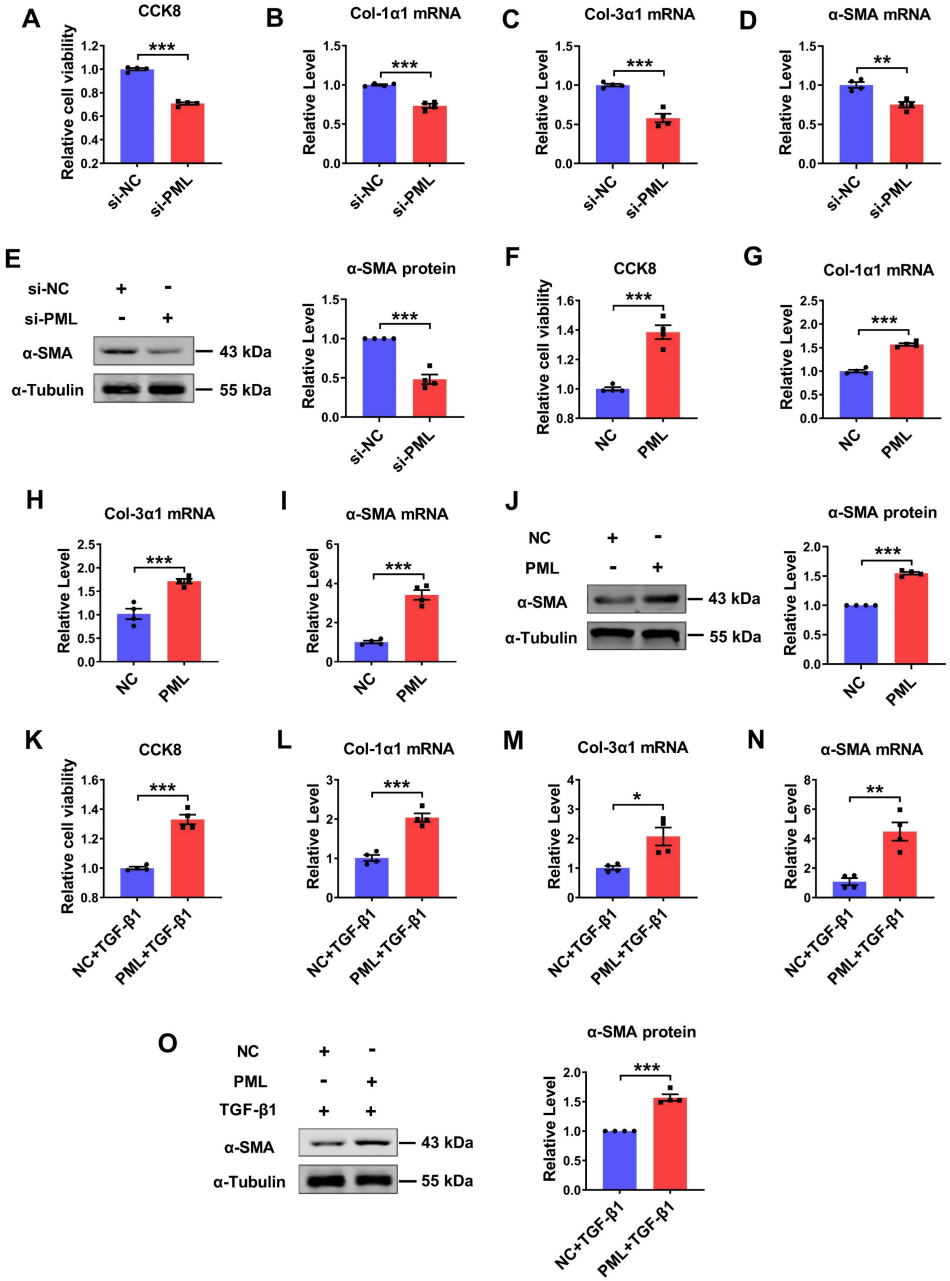
** Effects of PML knockdown or overexpression on cardiac fibroblasts. (A)** The effects of si-PML on cell viabilities in CFs were examined by the CCK8 assay. (n = 4). **(B-D)** qRT-PCR analysis of Col-1α1, Col-3α1 and α-SMA mRNA levels in CFs transfected with si-PML (n = 4). **(E)** Analysis of the expression of α-SMA by western blot after transfection with si-PML (n = 4). **(F)** The effects of PML overexpression on cell viabilities in CFs were examined by the CCK8 assay (n = 4). **(G-I)** qRT-PCR analysis of Col-1α1, Col-3α1 and α-SMA mRNA levels in CFs transfected with PML plasmid (n = 4). **(J)** Analysis of the expression of α-SMA by western blot after transfection with PML plasmid (n = 4). **(K)** The effects of PML overexpression on cell viabilities in TGF-β1 treated CFs were examined by the CCK8 assay (n = 4). **(L-N)** qRT-PCR analysis of Col-1α1, Col-3α1 and α-SMA mRNA levels in CFs transfected with PML plasmid and treated with TGF-β1 (n = 4). **(O)** Western blot quantification of α-SMA expression (n = 4). *P < 0.05, **P < 0.01, ***P < 0.001.

To explore the potential function of PML on cardiac fibroblasts, we performed loss- and gain-of-function experiments in cultured cardiac fibroblasts. The successful knockdown and overexpression of PML were confirmed both at the mRNA and protein levels (Fig. S2A-D). Silencing of endogenous PML expression resulted in a decrease in cell proliferation and Col-1α1, Col-3α1, α-SMA mRNA expressions (Fig. 2A-D), as well as attenuated the protein expression level of α-SMA in CFs (Fig. 2E). Thus, we further examined whether PML overexpression exerted pro-fibrotic effects on cardiac fibroblasts. The results showed that PML overexpression significantly increased cell proliferation and the mRNA expressions of Col-1α1, Col-3α1, α-SMA in CFs (Fig. 2F-I). Meanwhile, PML overexpression also augmented the expression of α-SMA protein (Fig. 2J). Similarly, overexpression of PML aggravated the activation of TGF-β1 on cardiac fibroblasts (Fig. 2K-O). These data suggest that PML impacts the activation of cardiac fibroblasts.

### P53 is essential for PML-NBs formation in cardiac fibroblasts

**Figure 3:**
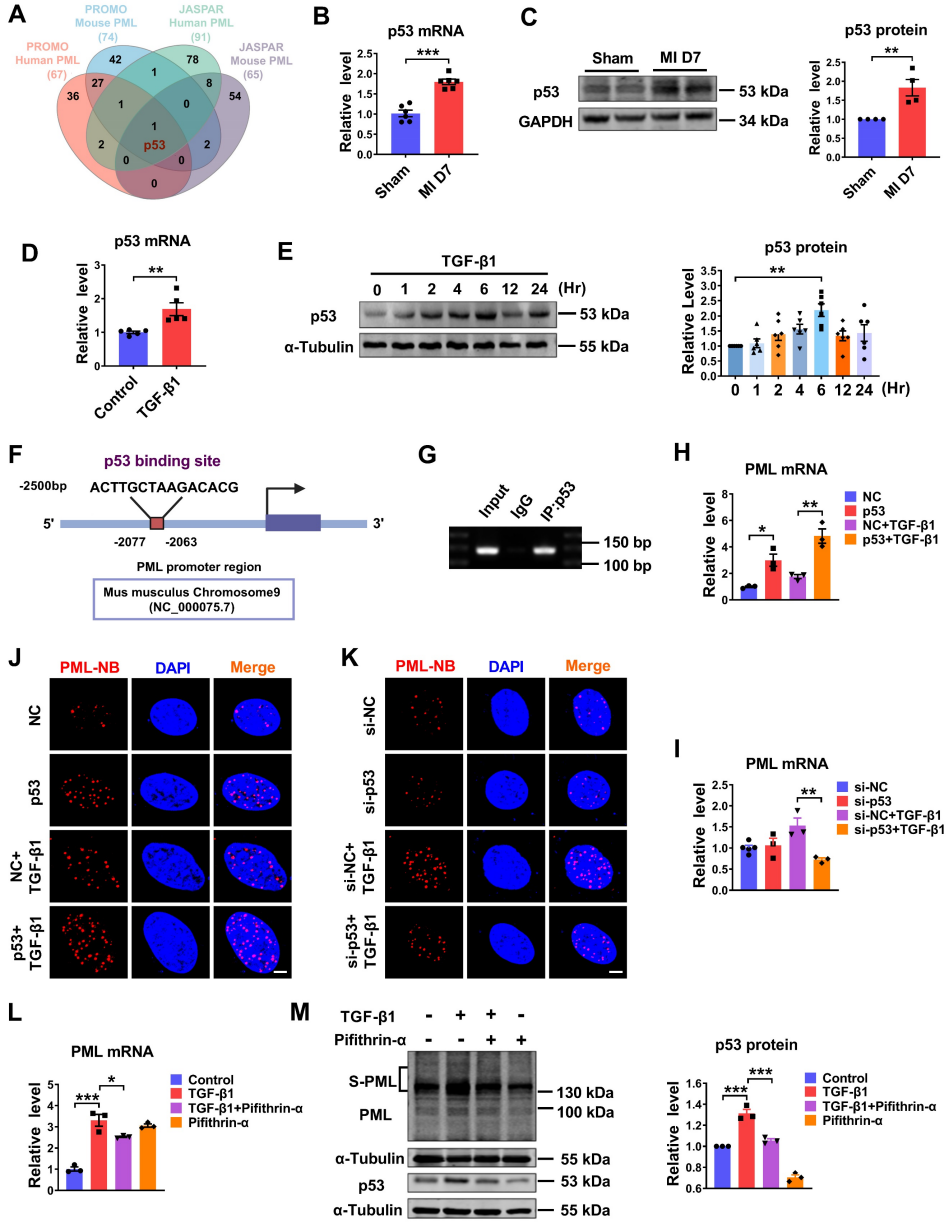
**P53 regulates PML gene expression and is essential for PML-NBs formation in response to TGF-β1. (A)** Venn diagram showing the transcription factor predicted by JASPAR and PROMO database. **(B)** The mRNA level of p53 in the infarct border zone of mouse left ventricular tissues at 7 days after MI operation was measured by qRT-PCR (n = 6). **(C)** Western blot analyses the quantification of p53 in the infarct border zone of mouse left ventricular tissues at 7 days after MI operation (n = 4). **(D)** qRT-PCR analysis of p53 mRNA levels in CFs after treatment with TGF-β1 (n = 5). **(E)** The protein level of p53 in CFs treated with TGF-β1 at the indicated time points (n = 6). **(F)** Conservative p53 DNA binding site in the PML promoter. JASPAR analysis showing the sequence of the potential binding site within the 2500 bp region upstream of the transcription start site of PML. **(G)** ChIP assay was used to determine the recruitment of p53 to the proximal promoter regions of PML in CFs (n = 3). **(H and I)** The expression of PML mRNA was detected by qRT-PCR after knockdown of p53 with si-p53, or overexpression of p53 by plasmid and treatment with or without TGF-β1 (n = 3). **(J and K)** PML-NBs in CFs transfected with p53 plasmid or si-p53 and treated with or without TGF-β1 were evaluated by immunofluorescence analysis. Scale bar, 5 μm (n = 3-5). **(L and M)** The mRNA level of PML and the protein levels of SUMOylated PML and p53 in CFs treated with TGF-β1 after the addition of pifithrin-α (10 μM) for 1 h (n = 3). *P < 0.05, **P < 0.01, ***P < 0.001.

The upregulation of PML mRNA and protein in response to fibrotic stimuli *in vivo* and *in vitro* suggests the potential transcriptional regulation of PML. According to the prediction of bioinformatics JASPAR (https://jaspar.genereg.net) and PROMO database (http://alggen.lsi.upc.es/cgi-bin/promo_v3/promo/promoinit.cgi?dirDB=TF_8.3), we found transcription factor candidates that might interact with the promoter region of PML and potentially regulate its expression. A Venn diagram showed that p53 was the potential transcription factor predicted by JASPAR and PROMO database (Fig. 3A). Accordingly, we detected the expression of p53 in fibrotic heart tissue induced by MI and in TGF-β1 treated cardiac fibroblasts. The result showed that p53 expression was elevated in the BZ of MI mice compared with the sham group (Fig. 3B-C). The mRNA level of p53 was significantly increased in TGF-β1 treated cardiac fibroblasts as well (Fig. 3D). In addition, we examined the kinetic patterns of p53 in TGF-β1 induced activation of CFs. As shown in Figure 3E, the level of p53 protein was rapidly increased by TGF-β1 at 2 h and reached its peak level at 6 h after TGF-β1 treatment. The phosphorylation level of p53 (phospho-p53) exhibited a similar kinetic pattern in TGF-β1 induced activation of CFs (Fig. S3A). Then, one putative binding site for p53 in the murine PML promoter region was revealed by motif analysis with JASPAR (Fig. 3F). To verify the transcription regulation of PML by p53 in CFs, we performed chromatin immunoprecipitation (ChIP) assay using the antibody against p53 in CFs. The result demonstrated that p53 protein was recruited to the putative p53 binding site on the proximal promoter region of PML. It indicates p53 has potential transcriptional activity to positively regulate PML transcription by direct binding to the PML promoter (Fig. 3G). Furthermore, both single overexpression of p53 and co-transfection of p53 with TGF-β1 in cardiac fibroblasts significantly increased PML mRNA expression (Fig. 3H), and vice versa, TGF-β1 failed to up-regulate PML expression after silencing p53 by si-p53 (Fig. 3I). Meanwhile, overexpression of p53 yielded a dramatic increase in PML-NB number, which was attenuated by knocking down p53 in cardiac fibroblasts with or without TGF-β1 treatment (Fig. 3J-K). Furthermore, we found that increased mRNA expression of PML by TGF-β1 was inhibited by pifithrin-α, a p53-specific inhibitor (Fig. 3L). Meanwhile, TGF-β1-mediated SUMOylation of PML was also abolished following the application of pifithrin-α (Fig. 3M). These results suggest the essential role of p53 as an upstream regulator of PML in cardiac fibroblasts.

### P53 is involved in cardiac fibroblasts activation upon TGF-β1 treatment

**Figure 4:**
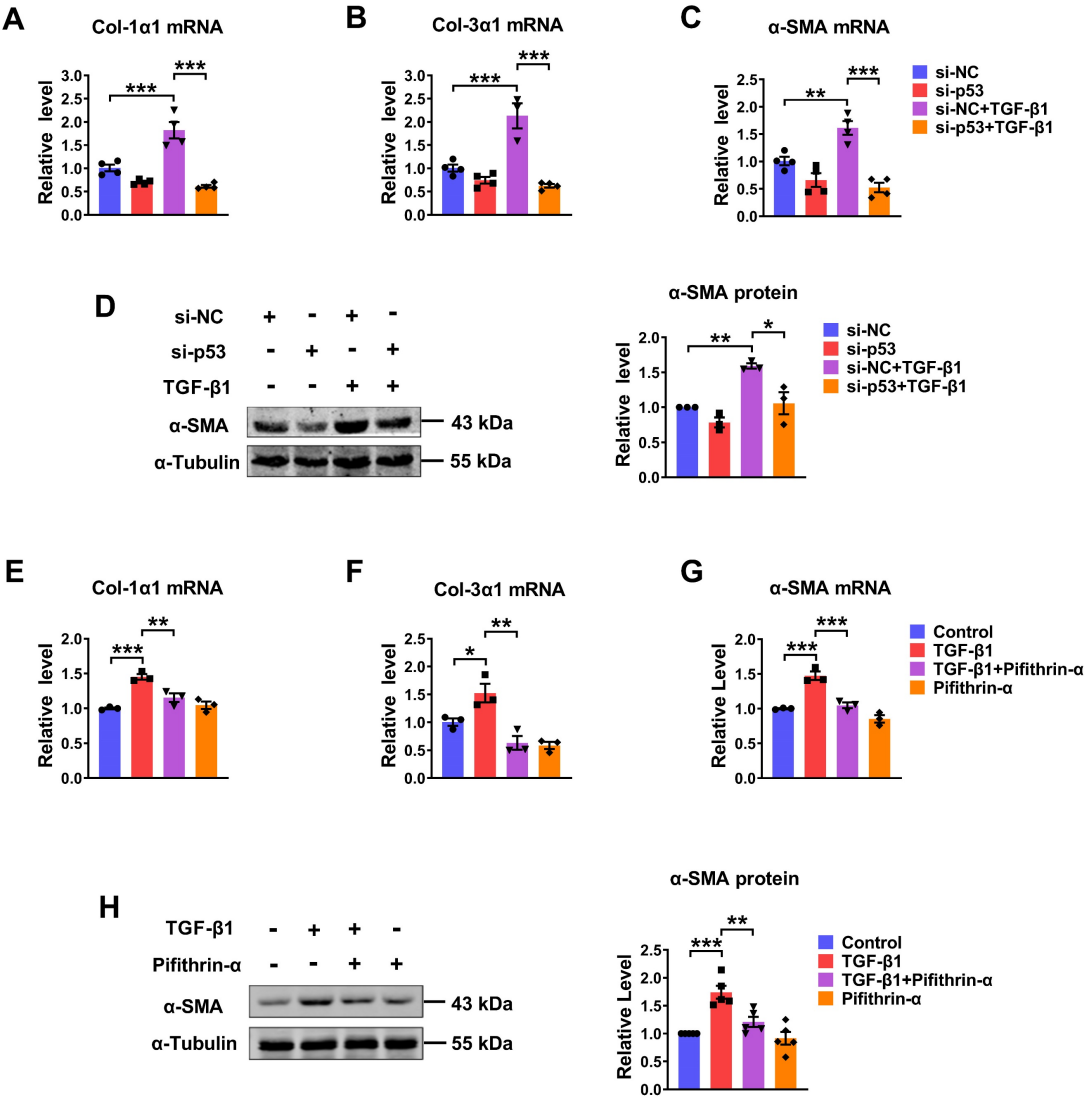
** Silencing/inhibition of p53 alleviated TGF-β1-induced cardiac fibrosis. (A-C)** qRT-PCR analysis of Col-1α1, Col-3α1 and α-SMA mRNA levels in CFs transfected with si-p53 and treated with or without TGF-β1 (n = 3-4). **(D)** Analysis of the expression of α-SMA by western blot (n = 3). **(E-G)** qRT-PCR showing the mRNA levels of Col-1α1, Col-3α1 and α-SMA in CFs treated with TGF-β1 after the addition of pifithrin-α (10 μM) for 1 h (n = 3). **(H)** Western blot quantification of α-SMA expression (n = 5). *P < 0.05, **P < 0.01, ***P < 0.001.

We further evaluated the potential role of p53 in the process of TGF-β1-induced cardiac fibrosis. Knocking down of endogenous p53 by si-p53 inhibited TGF-β1 induced increase of Col-1α1, Col-3α1, α-SMA mRNA expressions and protein level of α-SMA (Fig. 4A-D). Likewise, significantly increase of several fibrosis-related genes at both mRNA and protein levels in the TGF-β1 group was also prevented by pifithrin-α treatment (Fig. 4E-H). These results demonstrate that p53 is critical for TGF-β1-induced cardiac fibroblasts activation.

To investigate if p53 is indeed an important regulator of PML to regulate cardiac fibrosis, we further carried out experiments to reveal the relationship between PML and p53. As illustrated in Fig. S4A-E, pifithrin-α inhibited the increase of cell proliferation, the mRNA expressions of the pro-fibrotic factor, Col-1α1, Col-3α1, α-SMA, and the protein level of α-SMA in CFs treatment with TGF-β1, whereas this inhibitory effect was abolished by PML overexpression. It suggested that PML mediates the action of p53 to aggravate cardiac fibrosis.

### PML binds to p53 and stabilizes p53 in response to TGF-β1

**Figure 5:**
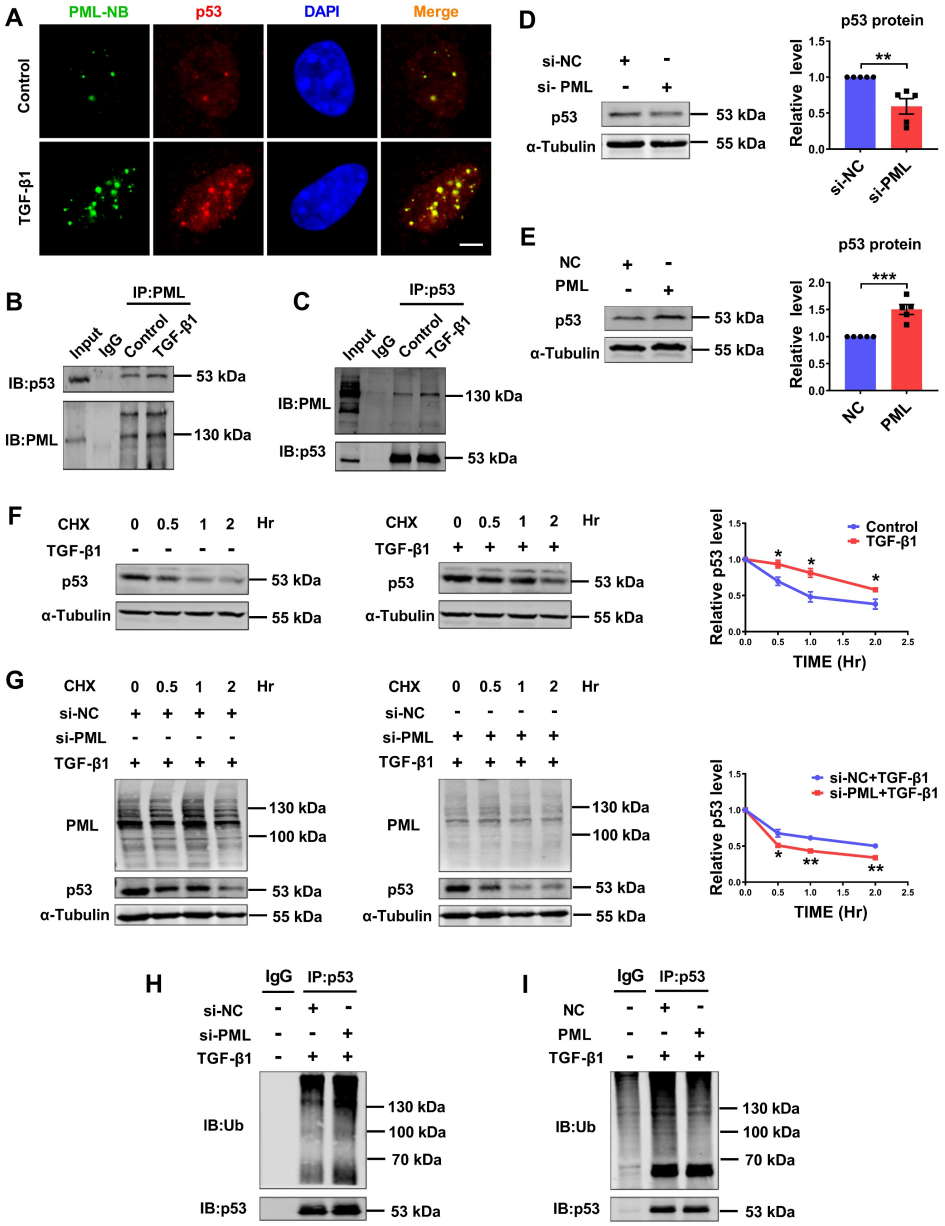
** PML stabilizes p53 by directly binding to p53 in response to TGF**-**β1. (A)** Co-localization of PML and p53 was detected by immunofluorescence analysis in CFs after treatment with TGF-β1. Scale bar, 5 μm (n = 5). **(B and C)** Representative immunoblots showing of co-immunoprecipitated PML/p53 complexes in CFs treated with TGF-β1. Extracts of cells were immunoprecipitated with either anti-PML or anti-p53 antibodies and then were precipitated with either antibody (n = 5). **(D)** Analysis of the expression of p53 by western blot after transfection of si-PML (n = 5). **(E)** Analysis of the expression of p53 by western blot after transfection of PML plasmid (n = 5). **(F)** P53 protein levels were measured at different time points in CFs treated with cycloheximide (CHX) (10 μg/ml), CHX plus TGF-β1 by western blot analysis (n = 3). **(G)** PML knockdown accelerates the degradation of p53. Twenty-four hours after transfection with si-PML, CFs were incubated with CHX plus TGF-β1 for the indicated times (n = 3). **(H and I)** The anti-p53 antibody was used to immunoprecipitate p53-ubiquitin immunocomplexes, followed by IB analysis using the anti-Ub antibody in CFs transfected with si-PML or PML overexpression plasmid after treatment with TGF-β1 (n = 3). *P < 0.05, **P < 0.01, ***P < 0.001.

We further investigated if there exists a regulatory loop between PML and p53 in the process of cardiac fibrosis. Immunofluorescence staining was employed to detect the interaction of PML with p53. As shown in Fig. 5A, TGF-β1 induced p53 accumulation in PML-NBs, as shown by the co-localization of p53 with PML. To further validate the immunofluorescence results, we conducted a Co-IP assay. As shown in Fig. 5B-C, the interaction of endogenous PML with endogenous p53, respectively, and this interaction was further strengthened when TGF-β1 was applied. The expression of p53 protein was consistent with that of PML after effectively silenced and overexpressed by si-PML and PML plasmid transfection in CFs (Fig. 5D-E), indicating that PML is required for the induction of p53 expression.

To further clarify whether PML is involved in the regulation of p53 stability, we measured the half-life of endogenous p53 protein in CFs chased with the translational inhibitor cycloheximide (CHX). We observed that the half-life of p53 was longer and more stable in TGF-β1-treated CFs compared to control cells (Fig. 5F). Silencing PML significantly shortened the half-life of p53 under TGF-β1 stimulation, suggesting PML knockdown promotes ubiquitination and degradation of p53 (Fig. 5G). Then, we investigated the effect of loss-and gain-of-function of PML on p53 ubiquitylation. The results showed that the ubiquitylation of p53 was markedly promoted by PML knockdown (Fig. 5H), however, dramatically suppressed by overexpression of PML in CFs treated with TGF-β1 (Fig. 5I). These data demonstrated that PML binds to and stabilizes p53 by attenuating its ubiquitin-dependent degradation in TGF-β1-treated cardiac fibroblasts.

### SUMOylated PML plays a regulatory role in p53 expression

**Figure 6:**
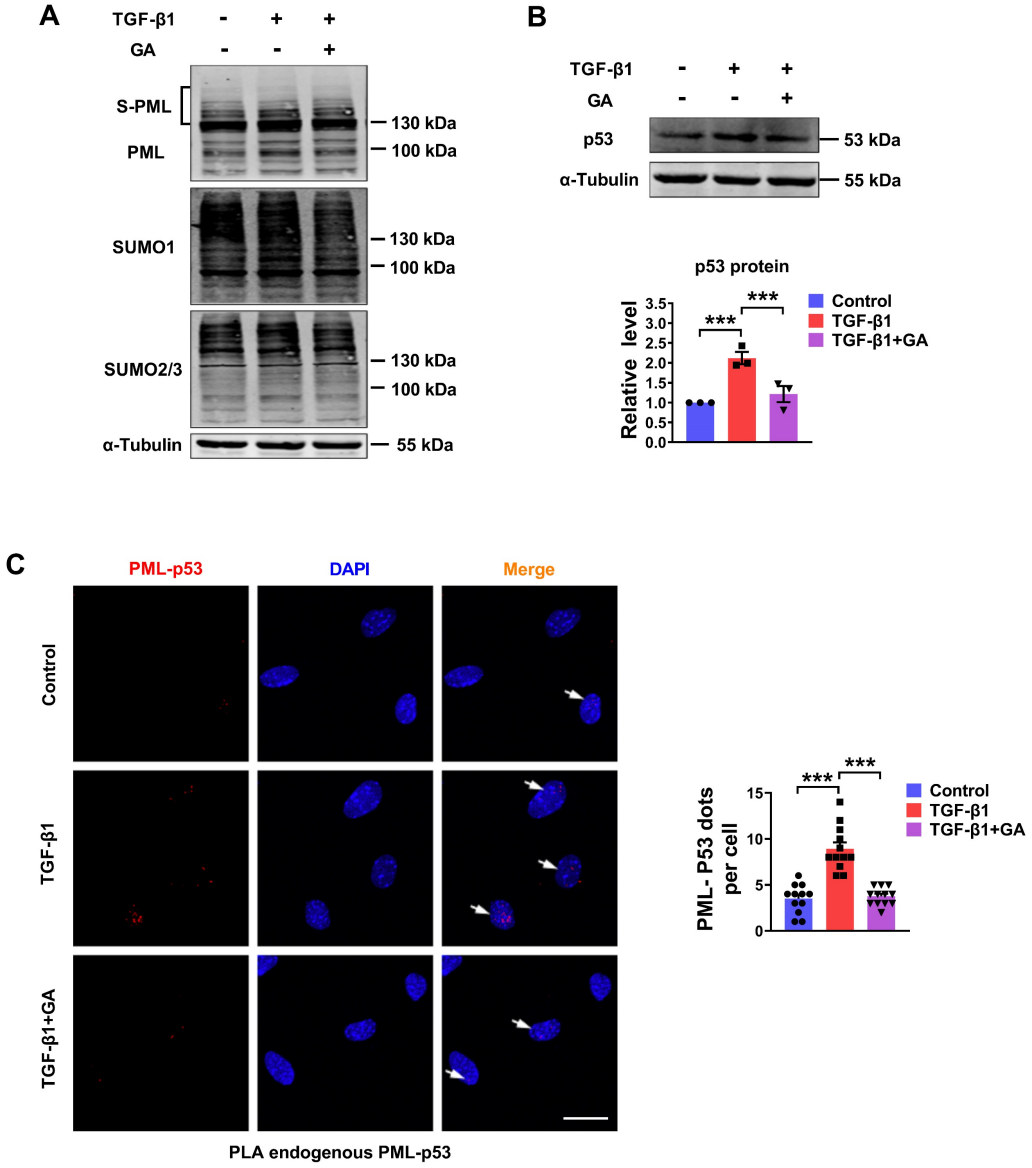
** Pharmacological inhibition of the SUMO pathway inhibits TGF-β1-induced PML/p53 interaction. (A and B)** The protein levels of SUMOylated PML, SUMO-1, SUMO-2/3 and p53 in CFs treated with TGF-β1 for 6 h with or without pretreatment with GA (10 μM) for 1 h (n = 3). **(C)** Interaction between PML and p53 (PML/p53, red) was assessed by PLA. Scale bar, 20 μm (n = 12). ***P < 0.001.

The SUMO-1 inhibitor, ginkgolic acid (GA), could bind directly to E1 and prevent E1-SUMO formation, leading to the inhibition of protein SUMOylation 15. To further explore whether the degradation process of p53 is involved in the PML mediated SUMOylation pathway, we detected SUMOylated PML, SUMO-1, SUMO-2/3 and p53 expression by western blot analysis in GA-pretreated cardiac fibroblasts. As shown in Fig. 6A-B, GA inhibited TGF-β1-induced SUMOylation of PML and expression of p53. Subsequently, we verified the interaction between endogenous PML and p53 by proximity ligation assay (PLA) in CFs. We observed a significant increase in the interactions of endogenous PML and p53 after TGF-β1 treatment. However, GA obviously abolished the interaction between PML and p53 (Fig. 6C). These results indicate that SUMOylated PML is critical for the interaction between PML and p53 and the subsequent p53 stabilization in TGF-β1 treated cardiac fibroblasts.

## Discussion

**Figure 7:**
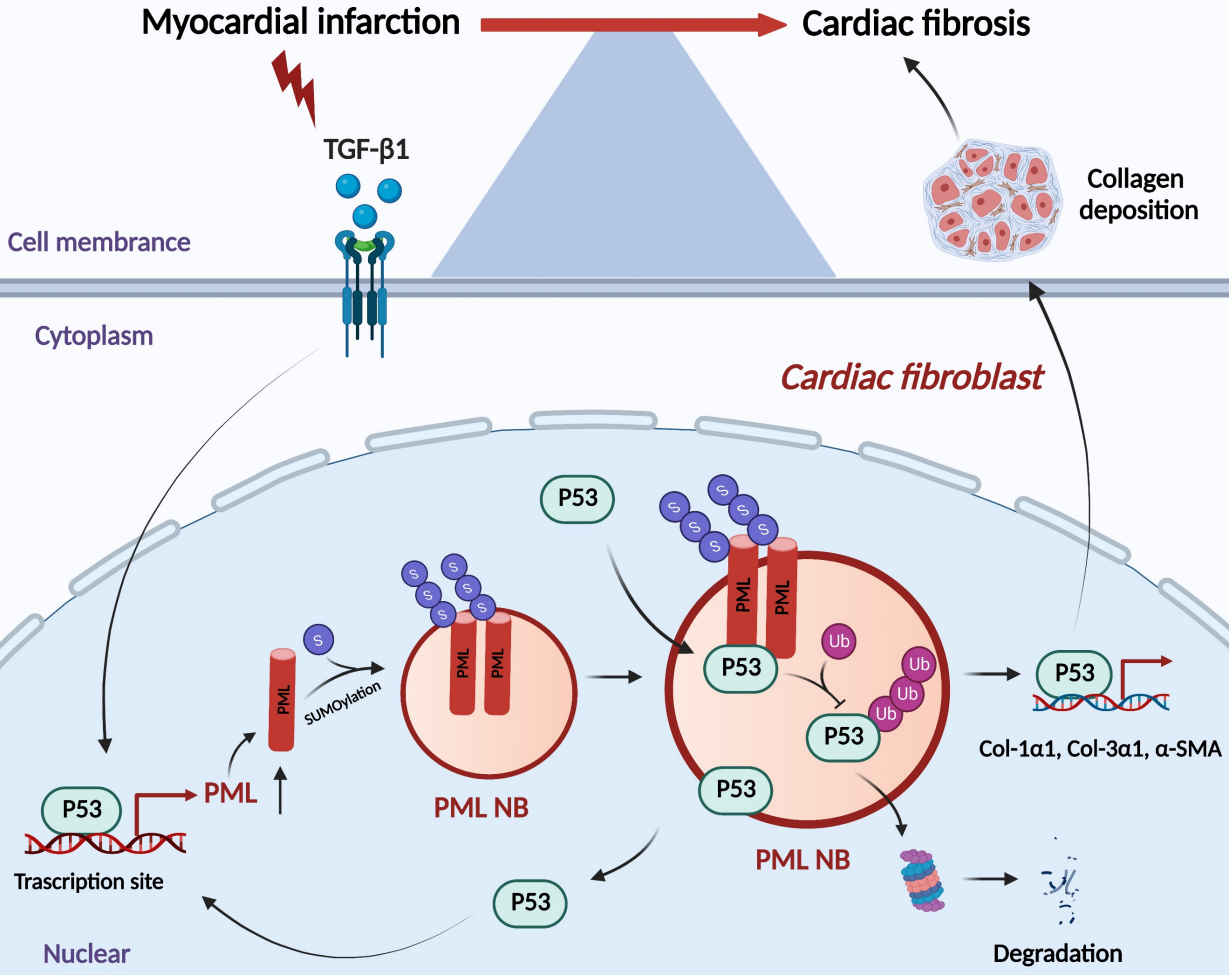
Schematic showing the proposed the PML/p53 positive feedback loop leading to cardiac fibroblasts activation.

The present study revealed that p53 is required for the transcriptional activation of PML, accumulation of PML protein and formation of PML-NBs in the process of myocardial fibrosis. As a consequence, PML stabilizes p53 through inhibiting ubiquitin-mediated proteasomal degradation, thus promoting p53-dependent profibrotic responses (Fig. 7).

Previous studies have indicated a critical role of SUMOylation/deSUMOylation balance for proper cardiac development. For instance, SUMO-1 and SUMO-2/3 levels were elevated during the compensated stage of hypertrophy and decreased rapidly following heart failure 10, 16. Consistent well with the notion that the SUMOylation of PML was increased in 1-week MI mice and subsequently declined sharply by 4 weeks. As a new stimulus, TGF-β1 was shown to induce both the accumulation of unmodified (95 kDa) and SUMO-modified (>130 kDa) PML. According to our previous study, TGF-β1 enhanced the SUMOylation of PML and recruitment of Pin1 to PML-NBs, leading to an increase in TGF-β1 expression in cardiac fibroblasts 17. A recent study showed that IFNα potentiated TGF-β-mediated conjugation of PML III and PML IV with SUMO, interacted with caspase-8 and recruited it within PML-NBs, resulting in enhanced induction of caspase-8-dependent apoptosis 18. Notably, the PML protein was identified as a critical TGF-β regulator. It has been reported that the cytoplasmic PML (cPML) functions as a cofactor for Smad2/3 and SARA (Smad anchor for receptor activation) and is required for Smad2/3 to associate with SARA and to activate TGF-β signals 19. The PCTA protein (PML competitor for TGIF association) can selectively compete with cPML for binding to TGIF. Therefore, the cytoplasm accumulates abundant cPML, which forms stable complexes with SARA and Smad2 and promotes the phosphorylation of Smad2 20. Additionally, the SUMO system can target components of the TGF-β1 signaling pathway, such as the TGF-β receptor I, Smad3 and Smad4 21. In this study, we found that forced expression of PML activated CFs proliferation, fibroblast-myofibroblast transition and then promoted collagen production and deposition in CFs, while knockdown of PML did the opposite. Therefore, our results inferred a possible connection between TGF-β1 signaling and PML in the pathogenesis of myocardial fibrosis.

Most studies concerning PML regulation have focused on its post-translational regulation, especially in terms of SUMOylation, phosphorylation, and ubiquitination pathways. There is, however, little knowledge about the transcriptional regulation of PML, especially in cardiovascular diseases. Here, we observed an increase in PML mRNA expression in cardiac fibrotic tissue from mice with myocardial infarction and in TGF-β1-induced cardiac fibroblasts. According to a bioinformatic analysis integrated with the JASPAR and PROMO databases, we determined that there is a potential binding site of p53 on the murine PML promoter. More recent studies have demonstrated crosstalk between p53 and TGF-β signaling. It has been reported that the phosphorylation and acetylation of p53 induced by TGF-β1 can facilitate the formation of p53/Smad3 complexes, which subsequently promote the transcription of profibrotic molecules 22. Here, we found that the expression levels of phospho-p53 and p53 were increased dramatically and then decreased gradually after TGF-β1 treatment. The observed increase in the active form of phosphorylated p53 and standard p53, demonstrated the activation of p53 in CFs. Then, we performed a ChIP experiment to explore a direct binding for p53 in the PML promoter, which provides evidence that PML is transcriptionally regulated by p53. However, it has been previously shown that PML transcription is enhanced by the p53 response element in the first intron region of PML during responses to oncogenes and DNA damage 23. Gain- and loss-of-function experiments show a positive correlation between PML transcription and p53 protein level. Moreover, p53 is required for TGF-β1-induced formation of PML-NBs. These data imply that PML as a potential p53 effector in CFs.

Upon oncogenic stress, DNA damage, or oxidative stress, PML functions as a p53 transcriptional co-activator through the recruitment of p53 to PML-NBs 24-27. In this study, we concluded that this reciprocal relationship between PML and p53 is a direct consequence of the binding of PML to p53 in CFs. Furthermore, studies have shown that PML may promote p53 stabilization by sequestering the p53 ubiquitin E3 ligase, Mdm2, in the nucleolus induced by various cellular stresses 28, 29. In the present study, we have identified PML plays a critical role in maintaining the stability of p53 and, therefore, as an essential determinant in cardiac fibrosis caused by the PML/p53 complex. Based on our results, we proposed that a positive feedback regulatory loop can promote cardiac fibrosis. We further investigate the correlation between PML and p53 in regulating cardiac fibrosis. PML-SUMO-1 conjugation and PML-NBs organization were reduced by GA pretreatment. With the decrease of PML-NBs, the expression of p53 was inhibited, and the PML/p53 interactions were abolished. These observations were consistent with our previous findings that GA ameliorated MI-induced fibrotic remodeling by inhibiting the SUMOylated PML/Pin1 axis 30.

Despite the robust findings of this study, there are some limitations that need to be discussed. P53 could be modified by conjugation to SUMO-1, which results in an increased transactivation ability of p53 31. Previous research showed that the SUMOylation of p53 and ERK5 by disturbed flow contributes to the formation of atherosclerotic plaques 32. So, whether there is an increase in p53 SUMOylation after TGF-β1 treatment and protecting p53 from degradation needs an in-depth investigation. In the nascent PML transcript, there are nine exons and multiple variants that can be roughly categorized as PML I to PML VII depending on the alternative splicing 33. Numerous studies evaluating the effects of PML on p53 function typically use PML IV. PML IV enhances the conjugation of p53 with SUMO-1, resulting in its stabilization and activation 34. These reports prompted us to explore whether PML IV mediates the TGF-β1-induced p53 activation in future work.

These data clearly demonstrate that transcriptional positive feedback loop involving p53 and PML is involved in the process of TGF-β1-induced cardiac fibrosis. In combination, these findings further our understanding regarding the role of PML in the pathogenesis of myocardial fibrosis and could lead the way to new therapeutic approaches and targets.

## Supplementary Material

Supplementary figures and tables.

## Abbreviations

ECM: extracellular matrix
PML: promyelocytic leukemia protein
TGF-β1: transforming growth factor-β1
SUMO: small Ubiquitin-like modifier
PML-NBs: PML nuclear bodies
GA: ginkgolic acid
MI: myocardial infarction
HF: heart failure
APL: acute promyelocytic leukemia
PTM: post‐translational modification
BZ: border zone
LV: left ventricular
S-PML: SUMO-modified PML
Co-IP: co-immunoprecipitation
CHX: cycloheximide
PLA: proximity ligation assay
SARA: Smad anchor for receptor activation
PCTA: PML competitor for TGIF association
cPML: cytoplasmic PML
DCM: dilated cardiomyopathy
ICM: ischemic cardiomyopathy
MMP-2: matrix metalloproteinase-2
CTGF: connective tissue growth factor
