# Supplementary material for: Crosstalk between PML and p53 in response to TGF-β1: A new mechanism of cardiac fibroblast activation
Source: Int J Biol Sci. 2023 Jan 22;19(3):994–1006. doi: 10.7150/ijbs.76214 (PMC9910009; doi:10.7150/ijbs.76214)
Supplement: Supplementary file 1 — Supplementary figures and tables. [file ijbsv19p0994s1.pdf]

1. Supplementary Figures

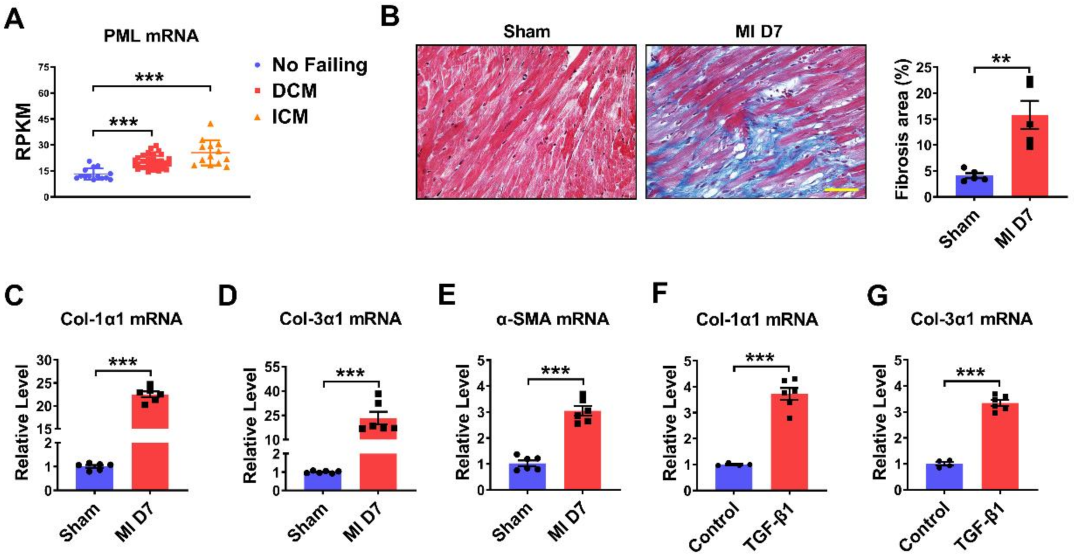

Supplementary Figure S1. Successful establishment of cardiac fibrosis model in vivo and in vitro.

(A) The transcriptional expression of PML was examined in RNA-sequencing data (GSE116250) including patients with normal myocardium (No Failing; n = 14), patients with dilated cardiomyopathy (DCM; n = 37), and patients with ischemic cardiomyopathy (ICM; n = 13). (**B**) Masson's trichrome staining of the LV sections of mouse hearts at 7 days after MI operation. Scale bar, 50 μm (n = 5). **(C-E)** qRT-PCR showing increased mRNA levels of Col-1α1, Col-3α1 and α-SMA in the infarct border zone of mouse left ventricular tissues at 7 days after MI operation (n = 6). **(F-G)** Following treatment with TGF-β1, Col-1α1 and Col-3α1 mRNA levels in CFs were examined by qRT-PCR (n = 4-6). $^{**}P < 0.01$, $^{***}P < 0.001$.

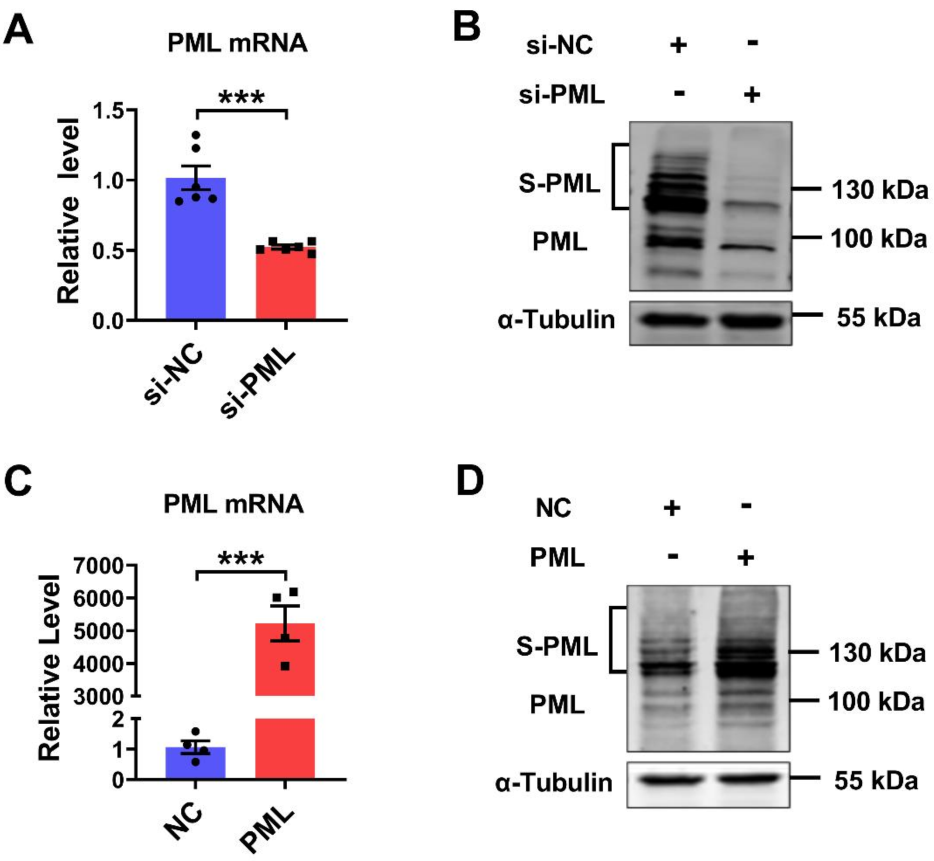

Supplementary Figure S2. The transfection efficiency of PML knockdown and overexpression in cardiac fibroblasts.

(A-D) qRT-PCR and western blot assay were used to verify PML knockdown or overexpression efficiency in CFs (n = 4-6). ***$P < 0.001$.

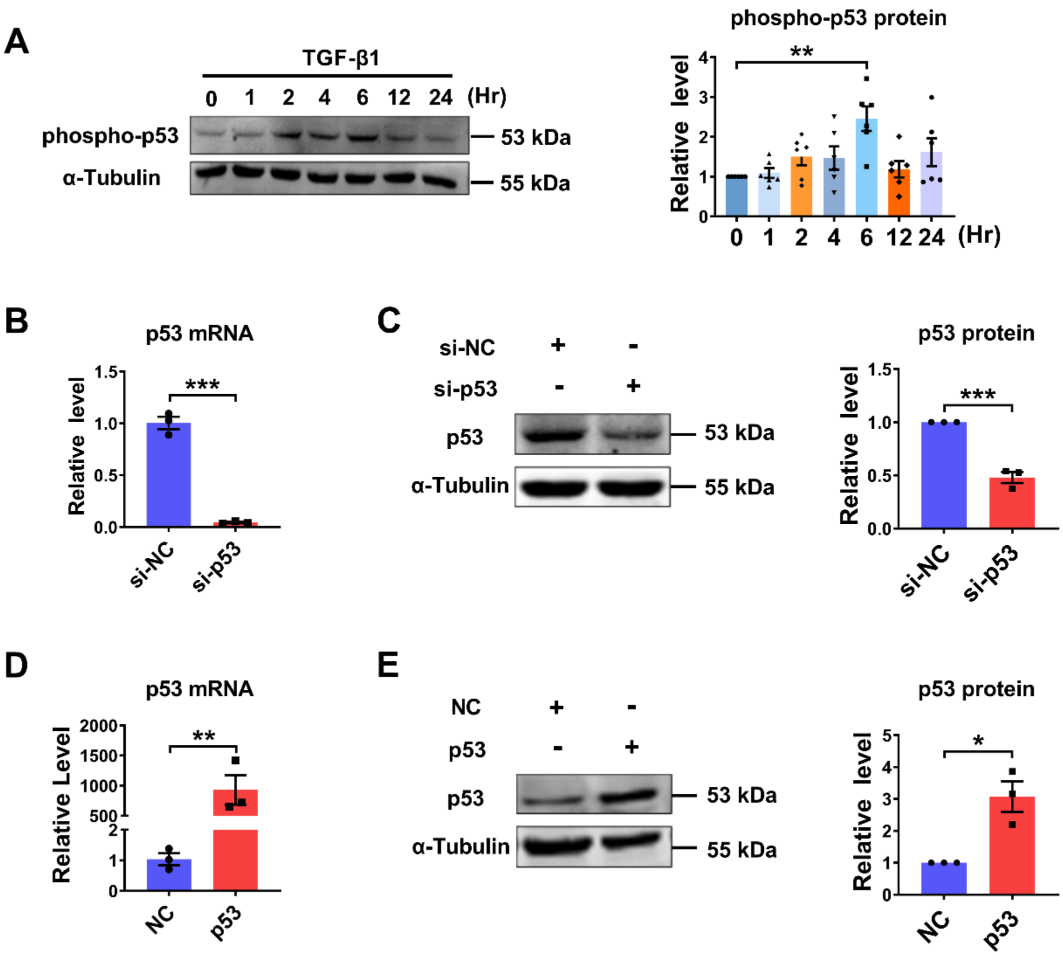

Supplementary Figure S3. The transfection efficiency of p53 knockdown and overexpression in cardiac fibroblasts.

(A) The protein level of phospho-p53 in CFs treated with TGF-β1 at the indicated time points (n = 6). **(B-E)** qRT-PCR and western blot assay were used to verify p53 knockdown or overexpression efficiency in CFs (n = 3). **P* < 0.05, ***P* < 0.01, ****P* < 0.001.

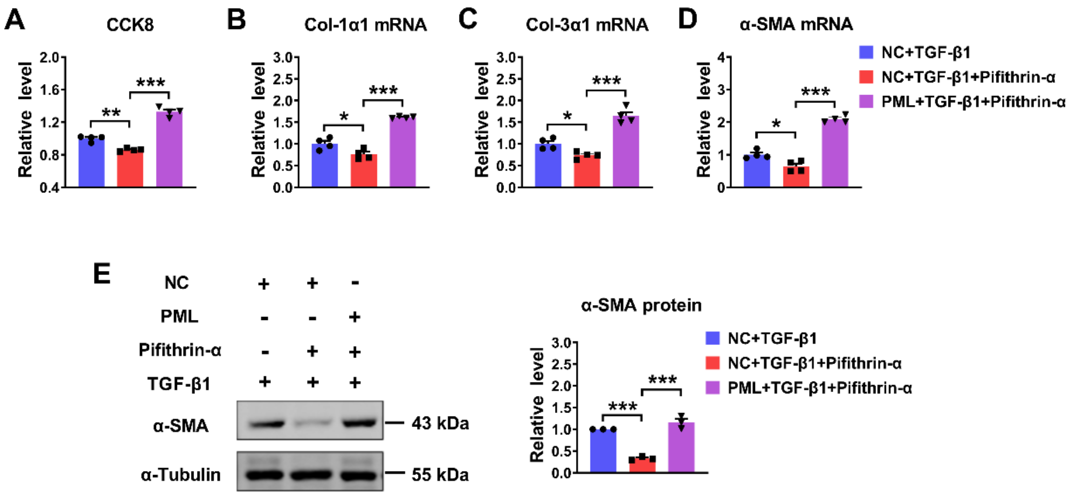

Supplementary Figure S4. Overexpression of PML cancelled the anti-fibrotic effect of p53 depletion.

(A) After transfection with PML plasmid, CFs were treated with or without Pifithrin-α in the presence of TGF-β1. Cell viabilities were examined by the CCK8 assay (n = 4). **(B-D)** qRT-PCR showing the mRNA levels of Col-1α1, Col-3α1 and α-SMA (n = 4). **(E)** Western blot quantification of α-SMA expression (n = 3). $^{*}P < 0.05$, $^{**}P < 0.01$, $^{***}P < 0.001$.

2. Supplementary Tables

Table S1 Sequences of the specific siRNAs

Gene	Sense (5'-3')	Antisense (5'-3')
si-PML	GCUGAUCUCCGCGACAUUTT	AAUUGUCGCGGAGAUCAGCTT
si-p53	CAUUUUCAGGCUUAUGGAATT	UUCCAUAAGCCUGAAAAUGTT

Table S2 Quantitative real-time PCR primer sequences of genes

Gene	Forward primer (5'-3')	Reverse primer (5'-3')
PML	CCTTTTCTTTTGACGGACCA	TGCAACACAGAGGCTTGGC
p53	TGGAGGAGTCACAGTCGGAT	CAGTGAGGTGATGGCAGGAT
Col-1α1	AAGAAGACATCCCTGAAGTCA	TTGTGGCAGATACAGATCAAG
Col-3α1	TTGGGATGCAGCCACCTTG	CGCAAAGGACAGATCCTGAG
TGF-β1	CCTGAGTGGCTGTCTTTTGACG	AGTGAGCGCTGAATCGAAAGC
α-SMA	CCCAGACATCAGGGAGTAATGG	TCTATCGGATACTTCAGCGTCA
GAPDH	GACAGCAGTTGGTTGGAGCA	TTGGGAGGGTGAGGGACTTC

Table S3 ChIP-qPCR primer sequences

Gene	Forward primer (5'-3')	Reverse primer (5'-3')
PML	CTCACAGACAGGGAAAA GCC	CAAGCAAGTAAACAAGC CCG
